# Endothelial to mesenchymal transition contributes to arsenic-trioxide-induced cardiac fibrosis

**DOI:** 10.1038/srep33787

**Published:** 2016-09-27

**Authors:** Yong Zhang, Xianxian Wu, Yang Li, Haiying Zhang, Zhange Li, Ying Zhang, Longyin Zhang, Jiaming Ju, Xin Liu, Xiaohui Chen, Peter V. Glybochko, Vladimir Nikolenko, Philipp Kopylov, Chaoqian Xu, Baofeng Yang

**Affiliations:** 1 Department of Pharmacology (the State-Province Key Laboratories of Biomedicine-Pharmaceutics of China, Key Laboratory of Cardiovascular Research, Ministry of Education), College of Pharmacy, Harbin Medical University, Harbin, 150081, China; 2 Institute of Metabolic Disease, Heilongjiang Academy of Medical Science, Harbin, 150086, China; 3 Center for Endemic Disease Control, Chinese Center for Disease Control and Prevention, Key Lab of Etiology and Epidemiology, Education Bureau of Heilongjiang Province & Ministry of Health (23618504), Harbin Medical University, Harbin, 150081, China; 4 The Research Center, Sechenov First Moscow State Medical University, Moscow, 119991, Russia; 5 Department of preventive and emergency cardiology, Sechenov First Moscow State Medical University, Moscow, 119991, Russia; 6 Department of Pharmacology and Therapeutics, Melbourne School of Biomedical Sciences, Faculty of Medicine, Dentistry and Health Sciences, The University of Melbourne, Melbourne, 3010, Australia

## Abstract

Emerging evidence has suggested the critical role of endothelial to mesenchymal transition (EndMT) in fibrotic diseases. The present study was designed to examine whether EndMT is involved in arsenic trioxide (As_2_O_3_)-induced cardiac fibrosis and to explore the underlying mechanisms. Cardiac dysfunction was observed in rats after exposure to As_2_O_3_ for 15 days using echocardiography, and the deposition of collagen was detected by Masson’s trichrome staining and electron microscope. EndMT was indicated by the loss of endothelial cell markers (VE-cadherin and CD31) and the acquisition of mesenchymal cell markers (α-SMA and FSP1) determined by RT-PCR at the mRNA level and Western blot and immunofluorescence analysis at the protein level. In the *in-vitro* experiments, endothelial cells acquired a spindle-shaped morphology accompanying downregulation of the endothelial cell markers and upregulation of the mesenchymal cell markers when exposed to As_2_O_3_. As_2_O_3_ activated the AKT/GSK-3β/Snail signaling pathway, and blocking this pathway with PI3K inhibitor (LY294002) abolished EndMT in As_2_O_3_-treated endothelial cells. Our results highlight that As_2_O_3_ is an EndMT-promoting factor during cardiac fibrosis, suggesting that targeting EndMT is beneficial for preventing As_2_O_3_-induced cardiac toxicity.

The first application of arsenic trioxide (As_2_O_3_) to patients with acute promyelocytic leukemia (APL) can be traced back to 1970s when the scholars in the Harbin Medical University conducted a small-scale clinical trial on it^1^,^2^. Since then, As_2_O_3_ has been widely used not only in clinical but also in basic research, shedding new light on the treatment of many other cancers^3^,^4^. However, the clinical use of As_2_O_3_ is restricted due to its cardiotoxicity, including long QT syndrome (LQTS) and torsades de pointes which can lead to sudden cardiac death^5^,^6^. A recent study revealed that As_2_O_3_-induced fibrosis in cardiac fibroblasts (CFs) is involved in the development of LQTS^7^. Cardiac fibrosis, a common pathological feature of many heart diseases, is associated with the disruption of normal cardiac structures and functions resulting from the excessive production and deposition of ECM in the myocardium^8^. Cardiac fibrosis plays a decisive role in the generation and development of heart disease and is involved in numerous forms of cardiovascular diseases including myocardial infarction, hypertension, and heart failure^9^,^10^,^11^.

Although accumulation of ECM proteins is an important characteristic in the fibrotic hearts, the mechanisms remain largely unknown. Myofibroblasts, characterized by α-smooth muscle actin (α-SMA) expression, are the primary determinant of cardiac fibrosis^12^. Myofibroblasts are mainly derived from resident fibroblasts, but recent studies revealed that they can also originate from transformation of vascular endothelial cells^13^. The transformation of vascular endothelial cells into myofibroblasts is a process known as endothelial-to-mesenchymal transition (EndMT), which was first identified in the embryonic development of the heart^14^. As a special form of epithelial to mesenchymal transition (EMT), EndMT is also involved in the development of cardiac fibrosis and other fibrotic diseases^15^,^16^,^17^,^18^,^19^. During the process of EndMT, endothelial cells lose their original phenotypes and eventually transform into a mesenchymal or myofibroblastic phenotypes with adoption of the migratory properties and acquisition of mesenchymal cell markers such as α-SMA and fibrotic-specific protein 1(FSP1)^17^. Most notably, many studies have concentrated on this newly recognized type of cellular transformation, and they found that EndMT is an important source of activated fibroblasts and myofibroblasts that actively participate in stimulus-induced cardiac fibrosis especially perivascular fibrosis^20^,^21^,^22^,^23^. However, the relationship between As_2_O_3_, EndMT and perivascular fibrosis, and also the underlying mechanisms remain unexplored.

In the present study, we explored the potential role of As_2_O_3_ in triggering EndMT to form cardiac fibrosis, and investigated the signaling pathway leading to EndMT in human aortic endothelial cells (HAECs).

## Results

### As_**2**_O_**3**_ impairs cardiac function in Wistar rats

**Figure 1:**
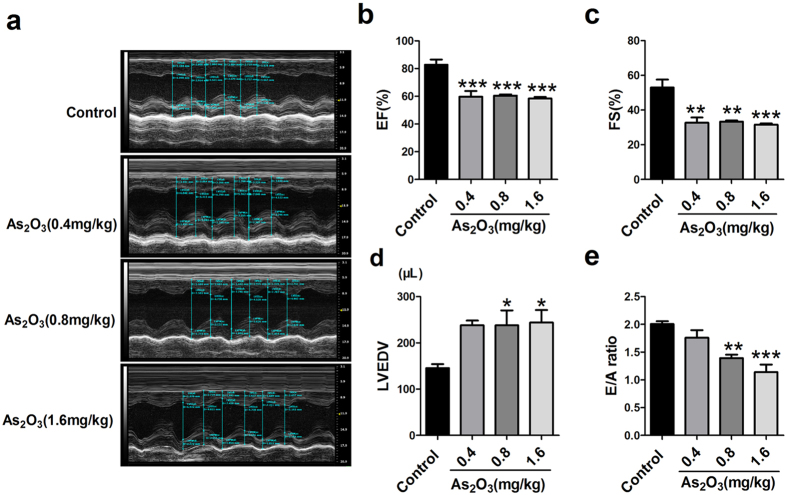
Effect of As_2_O_3_ on cardiac function determined by echocardiography. (**a**) Representative echocardiographic photos from M-mode. (**b**) Ejection fraction (EF) in each group. (**c**) Fraction shortening (FS) in each group. (**d**) Left ventricular end-diastolic volume (LVEDV) in each group. (**e**) Ratio of peak early diastolic ventricular filling velocity to peak atrial filling velocity (E/A). n = 4–5 rats in each group. *p < 0.05, **p < 0.01, ***p < 0.001 vs. Control.

The adverse effects of As_2_O_3_ on the heart have been reported in both clinical practice and basic research^24^,^25^. After two weeks of As_2_O_3_ administration, we performed echocardiographic measurements to evaluate the effect of As_2_O_3_ on cardiac function. The corresponding echocardiographic images in the control group and As_2_O_3_-treated groups (0.4 mg/kg, 0.8 mg/kg, 1.6 mg/kg) are shown in Fig. 1a. The ejection fraction (EF%) declined significantly from 82.83 ± 3.72% for control to 59.68 ± 4.21%, 60.57 ± 0.90% and 58.44 ± 1.06% for varying dosages of As_2_O_3_, respectively (Fig. 1b). Moreover, the fractional shortening (FS%) decreased dramatically from 53.03 ± 4.61% to 32.73 ± 2.96%, 33.33 ± 0.71% and 31.55 ± 0.73%, respectively (Fig. 1c). Additionally, we detected left ventricular end-diastolic volume (LVEDV) and ratio of peak early diastolic ventricular filling velocity to peak atrial filling velocity (E/A), which can be used as indicative markers of diastolic dysfunction. The results in Fig. 1d–e showed that LVEDV was elevated, while the E/A ratio was declined after exposure to As_2_O_3_. But this effect was only statistically significant at the dosages of 1.6 mg/kg and 0.8 mg/kg. The detailed data for end-systolic and end-diastolic volumes and other additional parameters were also provided (Supplementary Table 2). All of these data indicated that cardiac dysfunction appeared when the rats received As_2_O_3_ treatment.

### As_2_O_3_ induces cardiac fibrosis

**Figure 2:**
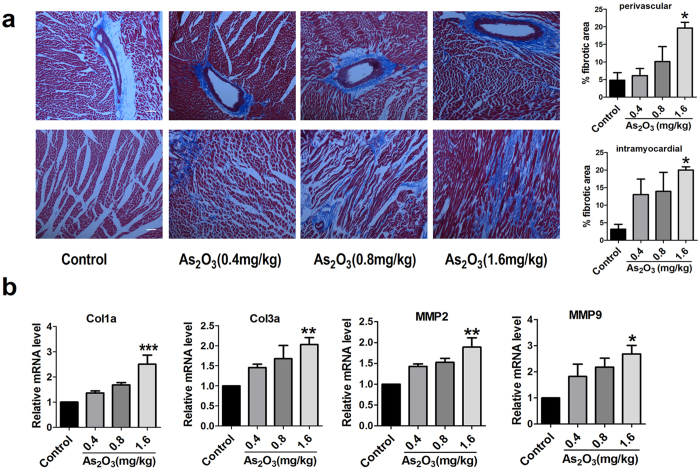
Collagen production and fibrotic gene expression in As_2_O_3_-treated animal models. (**a**) Masson trichrome staining for collagen deposition in cardiac cross-sectional parts. Collagen is indicated as blue areas, scale bar = 200 μm. (**b**) mRNA expression level of the fibrosis-related genes Col1a, Col3a, mmp2, mmp9. *p < 0.05, **p < 0.01, ***p < 0.001 vs. Control. Graph bars represent mean ± SEM, n = 3–5.

Excessive production and deposition of extracellular matrix (ECM) proteins are the primary characteristic of cardiac fibrosis. To verify whether As_2_O_3_ could induce cardiac fibrosis, Masson’s trichrome staining and transmission electron microscopy were performed. Collagen production and deposition increased markedly in the As_2_O_3_-treated groups compared with the control group in both perivascular region and intramyocardial region (Fig. 2a). Meanwhile, the transmission electron microscopy images demonstrated that exposure to As_2_O_3_ (1.6mg/kg) significantly increased ECM deposition compared with the control group (Supplementary Figure 1). Additionally, qRT-PCR results revealed that the expression of pro-fibrotic genes including Col1a, Col3a, mmp2 and mmp9 was upregulated in the As_2_O_3_-treated groups (Fig. 2b). However, it should be noted that significant effects of As_2_O_3_ on cardiac fibrosis were seen only at the high-dose group, even though the same trend of changes was also observed with lower dosages.

### EndMT is present in As_**2**_ **O**_3_-induced cardiac fibrosis

**Figure 3:**
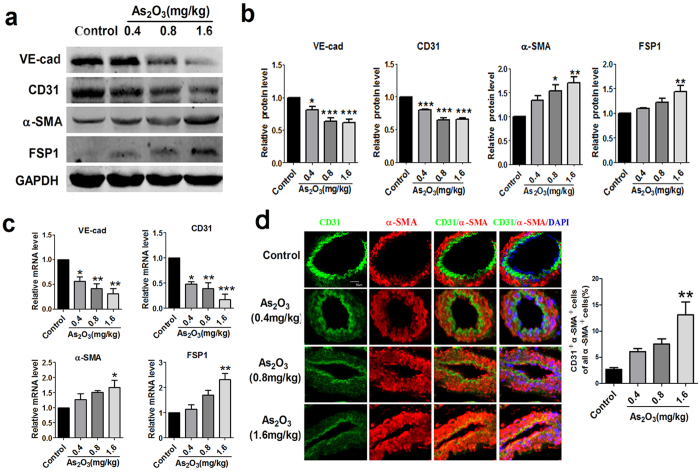
EndMT in As_2_O_3_-induced cardiac fibrosis. (**a**) Representative protein bands of endothelial markers VE-cadherin (VE-cad) and CD31 and mesenchymal markers α-smooth muscle actin (α-SMA) and fibroblast-specific protein-1 (FSP1) in cardiac tissues by western blotting. (**b**) Analysis of western blotting results normalized to glyceraldehyde phosphate dehydrogenase (GAPDH). (**c**) Quantitative real-time PCR experiments for relative mRNA levels of VE-cad, CD31, α-SMA, FSP1. No significant difference was observed between the different dosages groups. *p < 0.05, **p < 0.01, ***p < 0.001 vs. Control. Data are expressed as mean ± SEM, n = 3–5. (**d**) Double immunofluorescence labeling of CD31 (green) and α-SMA (red) in As_2_O_3_ treated hearts, nuclei are stained with DAPI (blue). Yellow indicates co-localization of CD31 with α-SMA expression in vessels. Scale bar = 30 μm. **p < 0.01 vs. Control, n = 3.

Accumulating evidence suggests that EndMT is an important contributor to cardiac fibrosis^15^. Therefore, we investigated whether As_2_O_3_ induced cardiac fibrosis was mediated by EndMT. As shown in Fig. 3a,b, the downregulation of endothelial–specific markers (VE-cadherin and CD31) and upregulation of mesenchymal markers (α-SMA and FSP1) were observed in hearts of As_2_O_3_ treated rats. However, these changes were only statistically significant at the high-dose group, but not at the two lower dose groups, especially for the expression of mesenchymal markers. Meanwhile, we obtained similar results using qRT-PCR analysis (Fig. 3c). Moreover, the expression of typical transcriptional factors (Snail, Twist, Slug) for EndMT was also markedly increased at the high-dose group (Supplementary Figure 2). We next performed double immunofluorescence staining of CD31 (green) and α-SMA (red) for EndMT and observed the co-localization of CD31 and α-SMA in myocardial sections of rats with the high-dose As_2_O_3_ (Fig. 3d). All of these results suggest that EndMT might be involved in As_2_O_3_-induced cardiac fibrosis.

### As_**2**_O_3_ treatment triggers EndMT in HAECs

**Figure 4:**
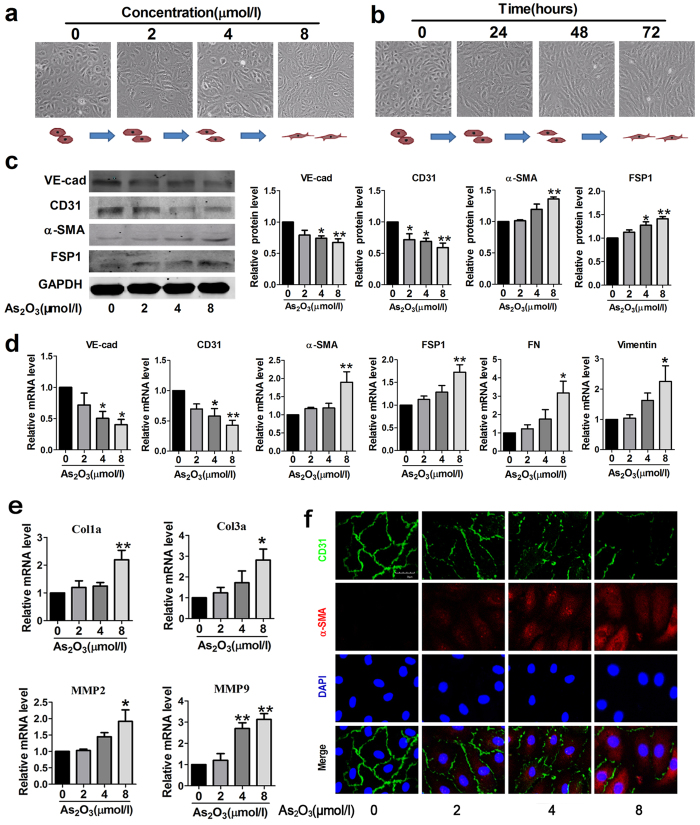
As_2_O_3_ triggers EndMT in human aortic endothelial cells (HAECs). (**a**) Morphological changes of endothelial cells exposed to different concentrations of As_2_O_3_. (**b**) Morphological changes of endothelial cells treated with As_2_O_3_ (2 μmol/l) for 0, 24, 48 or 72 hours. (**c**) Western blotting results for relative protein levels of VE-cad, CD31, α-SMA and FSP1 in As_2_O_3_-treated HAECs. GAPDH was used as an internal control. (**d**) Relative expression levels of endothelial markers (VE-cad, CD31) and mesenchymal markers (α-SMA, FSP1, FN and Vimentin) were compared by qRT-PCR between control groups and As_2_O_3_-treated groups. FN, fibronectin. (**e**) Relative mRNA levels of fibrosis-related genes Col1a, Col3a, mmp2 and mmp9. (**f**) Representative confocal microscopy images showing staining of endothelial marker CD31 and mesenchymal marker α-SMA in As_2_O_3_-treated HAECs. Scale bar = 30 μm. *p < 0.05, **p < 0.01 vs. untreated condition (0 μmol/l As_2_O_3_). No significant difference was observed between the different concentrations groups. Data are represented as mean ± SEM, n = 3–5.

To further elucidate the relationship between As_2_O_3_ and EndMT, we used HAECs for our subsequent *in vitro* experiments. After treatment with varying concentrations of As_2_O_3_ for 24 h, the endothelial cells underwent a morphological transformation to fibroblast-like spindle-shaped phenotype (Fig. 4a). However, we should note that the morphological change of endothelial cells at the concentration of 2 μmol/l was not as obvious as that at 4 μmol/l and 8 μmol/l. When we prolong the treatment time to 48 h and 72 h, this change became more remarkable (Fig. 4b). Besides, As_2_O_3_ reduced the expression of endothelial cell markers (VE-cadherin and CD31) and augmented the expression of mesenchymal markers (α-SMA and FSP1) in a concentration-dependent manner as indicated by western blotting results (Fig. 4c). Similar results were obtained with qRT-PCR analysis (Fig. 4d). Additionally, we detected two other mesenchymal markers fibronectin (FN) and vimentin and found that the expression of FN and vimentin had the same trend as the expression of α-SMA and FSP1 (Fig. 4d). Meanwhile, the mRNA levels of fibrotic-related genes, including Col1a, Col3a, mmp2 and mmp9, were markedly increased (Fig. 4e). The results from double immunofluorescence staining further confirmed that endothelial cells with As_2_O_3_ treatment underwent EndMT with a decrease for the membrane staining of CD31 and increase for the cytoplasm staining of α-SMA (Fig. 4f). Taken together, we have demonstrated that As_2_O_3_ could elicit EndMT in HAECs.

### The AKT/GSK-3β/Snail pathway is activated in As_2_O_3_-mediated EndMT

**Figure 5:**
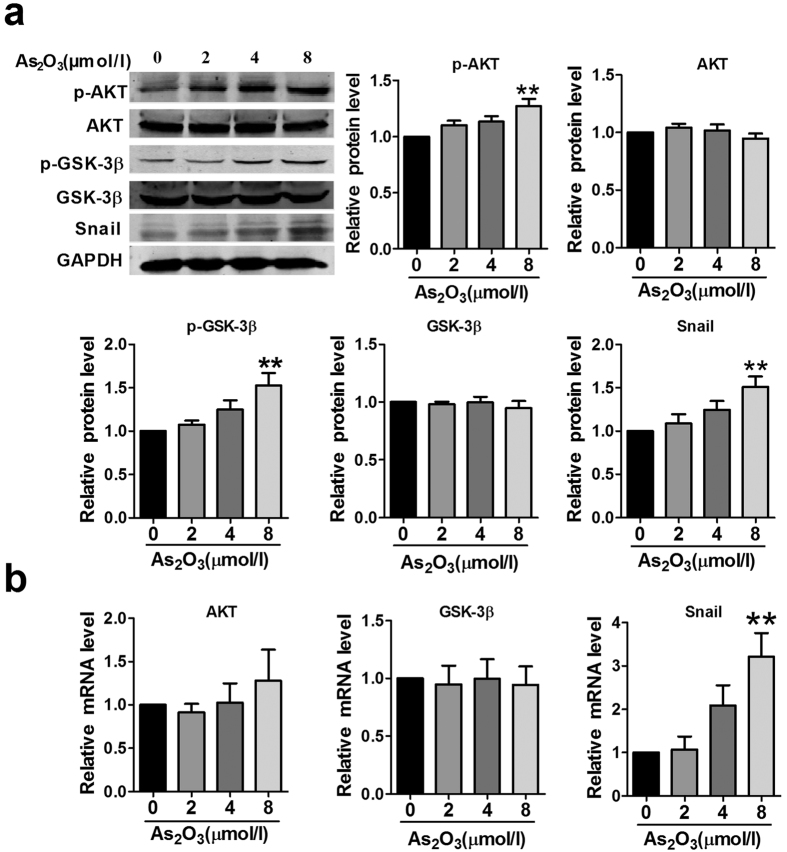
As_2_O_3_ activates the AKT/GSK-3β/Snail pathway. (**a**) HAECs were treated with or without As_2_O_3_, and the expression of Snail and the activation of AKT and GSK-3β were determined by western blotting. (**b**) Relative mRNA expression level of AKT, GSK-3β and Snail in different groups. **p < 0.01 vs. untreated condition (0 μmol/l As_2_O_3_). Data are expressed as mean ± SEM, n = 3–5.

Multiple signaling pathways are involved in the process of EndMT, such as the TGF-β pathway, Notch pathway, and PI3K/AKT pathway^26^,^27^,^28^,^29^. The AKT/GSK-3β/Snail pathway has been proposed to play an important role in inducing EMT^30^,^31^. Because EndMT is a special form of EMT, here we hypothesized that As_2_O_3_ may induce EndMT via the AKT/GSK-3β/Snail pathway. As expected, upon treatment with a high concentration of As_2_O_3_ (8 μmol/l) for 24 h, phosphorylation of AKT and GSK-3β was dramatically increased and Snail expression was significantly enhanced in HAECs. The total protein and mRNA levels of AKT and GSK-3β were unaffected by As_2_O_3_ at concentrations of 2, 4 and 8 μmol/l (Fig. 5a,b). To further demonstrate the phenomenon of AKT/GSK-3β/Snail signaling activation *in vivo*, we double-stained CD31/p-AKT, CD31/p-GSK-3β and CD31/Snail in heart tissues. The results in Supplementary Figure 3 showed that the expression of p-AKT, p-GSK-3β and Snail were at a higher level in endothelial cells of myocardial sections from As_2_O_3_-treated rats, which suggested that the AKT/GSK-3β/Snail signaling pathway is also activated *in vivo*.

### **Blocking As**_**2**_ **O**_**3**_ **-**induced phosphorylation of AKT inhibits EndMT in HAECs

**Figure 6:**
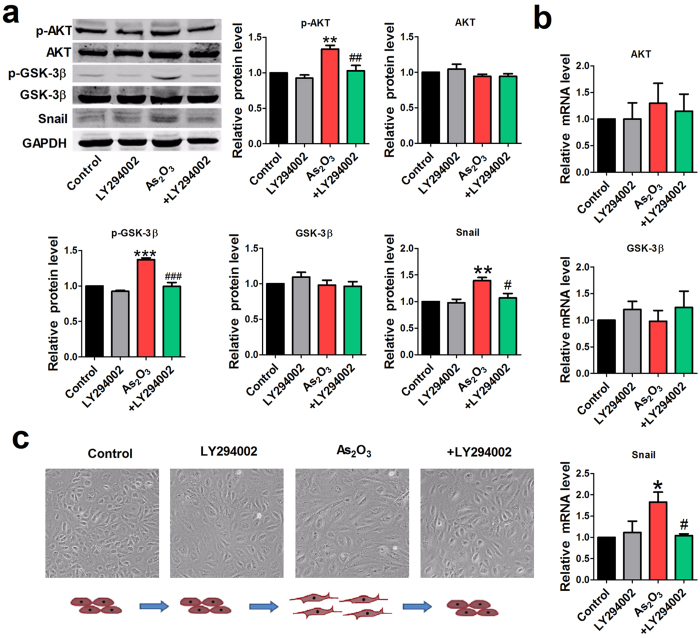
Effects of the PI3K inhibitor LY294002 on the AKT/GSK-3β/Snail pathway and EndMT phenotypes in HAECs. (**a**) Before treatment with As_2_O_3_ (8 μmol/l), HAECs were pretreated with LY294002 for 2 h. The activation of AKT and GSK-3β and the expression of snail were analyzed by western blotting. +LY294002 indicates the co-application of LY294002 and As_2_O_3_ (8 μmol/l). (**b**) Relative mRNA level of AKT, GSK-3β and snail in different groups. *p < 0.05, **p < 0.01, ***p < 0.001 vs. Control. ^#^p < 0.05, ^##^p < 0.01, ^###^p < 0.001 vs. As_2_O_3_. Data are shown as mean ± SEM, n = 3–5. (**c**) Effects of LY294002 on the morphologic phenotype of HAECs.

To clarify the role of the AKT/ GSK-3β/Snail pathway in As_2_O_3_-induced EndMT, LY294002 (an inhibitor of PI3K/AKT) was used to treat HAECs prior to As_2_O_3_ treatment. As shown in Fig. 6a, the expression levels of phosphorylated AKT, phosphorylated GSK-3β and Snail were markedly increased after As_2_O_3_ treatment, whereas these effects were prevented upon pre-treatment with LY294002 in combination with As_2_O_3_ in HAECs. But the protein and mRNA level of AKT and GSK-3β were not affected by LY294002 (Fig. 6a,b). Notably, we found that pre-treatment with LY294002 abrogated the morphological conversion of HAECs induced by As_2_O_3_ (Fig. 6c). While LY294002 applied alone did not exert much influence on cells phenotype in comparison to control group.

**Figure 7:**
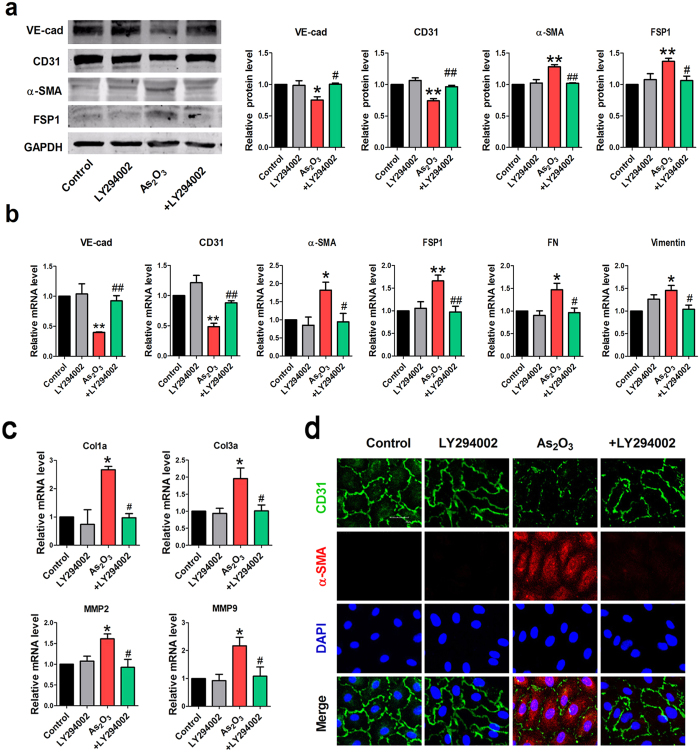
PI3K inhibitor LY294002 represses As_2_O_3_-induced EndMT in HAECs. (**a**) Western blotting results for relative protein levels of VE-cad, CD31, α-SMA and FSP1 in different groups. +LY294002 indicates the co-application of LY294002 and As_2_O_3_ (8 μmol/l). (**b**) Relative mRNA expression levels of VE-cad, CD31, α-SMA, FSP1, FN and Vimentin in each group by qRT-PCR analysis. (**c**) Effects of LY294002 on the mRNA expression of fibrosis-related genes Col1a, Col3a, mmp2, mmp9. *p < 0.05, **p < 0.01 vs. Control. ^#^p < 0.05, ^##^p < 0.01 vs. As_2_O_3_. Data are shown as mean ± SEM, n = 3–5. (**d**) Immunofluorescence analysis for the expression levels of CD31 and α-SMA in Control, LY294002, As_2_O_3_ and As_2_O_3 _+ LY294002 groups. Scale bar = 30 μm.

We also detected EndMT-associated markers in the condition of AKT pathway inhibition. Western blotting analysis showed that LY294002 effectively prevented the downregulation of endothelial markers and upregulation of mesenchymal markers by As_2_O_3_ (Fig. 7a). Consistently, the qRT-PCR results revealed that the As_2_O_3_-induced reduction of endothelial markers and augmentation of mesenchymal markers and fibrosis-related gene expression were attenuated by pre-treatment with LY294002 (Fig. 7b,c). Moreover, the immunostaining assay confirmed the inhibitory effects of the PI3K inhibitor (LY294002) on EndMT (Fig. 7d).

## Discussion

**Figure 8:**
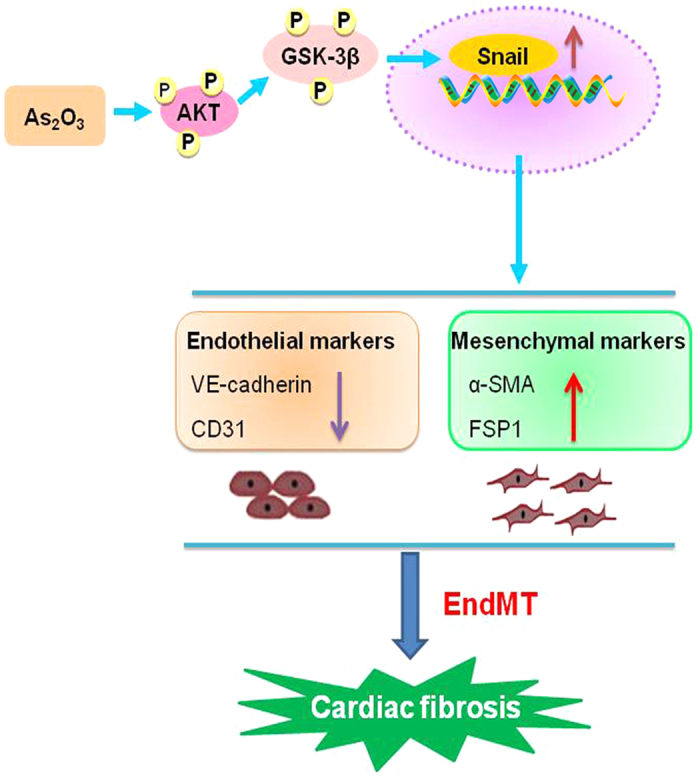
A schematic diagram revealing the underlying mechanisms of As_2_O_3_ induced EndMT and cardiac fibrosis.

In the present study, we made an effort to understand the potential role of EndMT in As_2_O_3_-induced cardiac fibrosis. First, *in vivo* study showed that EndMT was present in the progression of cardiac fibrosis and cardiac dysfunction. Second, *in vitro* study confirmed that As_2_O_3_ could result in EndMT in HAECs, and this process was partly mediated by the AKT/GSK-3β/Snail pathway. The proposed mechanism is illustrated in Fig. 8.

Cardiac fibrosis, as an adaptive response or repair mechanism, can be defined as excessive deposition of ECM components in the cardiac interstitium. Studies have found that cardiac fibrosis leads not only to heart failure but also to fatal arrhythmia^32^,^33^, which can be attributed to the reduction of cardiac contractility and compliance. Therefore, we have a reason to believe that myocardial fibrosis is responsible for the induction of cardiac toxicity in APL patients receiving As_2_O_3_ therapy or in conditions of arsenic exposure. Clinically, As_2_O_3_ is administrated intravenously at dosages of 10 mg/d for adults and 0.16 mg/kg for children^34^,^35^. Pharmacokinetic studies showed that the mean peak level of As_2_O_3_ in plasma is 6.85 μmol/l^34^. The dosages of As_2_O_3_ tested in our *in vivo* studies (0.4, 0.8 and 1.6 mg/kg) fall right within this clinical range and are also in accordance with the published studies relevant to the cardiotoxicity of As_2_O_3_^7^,^36^. Moreover, the concentration range of As_2_O_3_ (2~8 μmol/l) used in our *in vitro* study encompasses the clinically relevant concentrations.

Traditionally, the activation and proliferation of resident fibroblasts are considered a key source of myofibroblasts in cardiac fibrosis. Many anti-fibrotic agents are focused on inhibiting cardiac fibroblast proliferation and collagen production^37^,^38^. Recently, EndMT was found to be as an alternative origin of myofibroblasts and to play an increasingly important role in the development of cardiac fibrosis. A study by Zeisberg E.M. *et al*. first demonstrated the contribution of EndMT to the total pool of cardiac fibroblasts. They used fate mapping method to identify the origin of cardiac fibroblasts and found that endothelial cells are responsible for the emergence of fibroblasts^15^. Another study by Widyantoro *et al*. showed that endothelial cell-derived endothelin-1 (ET-1) could stimulate the EndMT process and promote diabetes mellitus–induced cardiac fibrosis^23^. Moreover, Rining Tang and colleagues revealed that high glucose-induced EndMT is mediated by angiotensin II and this process could be inhibited by Irbesartan^39^. Furthermore, Ignacio Montorfano *et al*. found that oxidative stress could induce the conversion of endothelial cells into myofibroblasts through the TGF-β dependent pathway^40^. All of these findings underline the prevalence of EndMT in cardiac fibrosis.

EndMT is a process of cellular transdifferentiation with similarities to epithelial-to-mesenchymal transition (EMT) in which endothelial cells lose their cell-cell junctions, change their morphology into a mesenchymal or myofibroblastic phenotype, express mesenchymal cell markers and gain migratory or contractile properties. Here we performed confocal microscopy analysis to assess the co-localization of CD31 (endothelial marker) and α-SMA (mesenchymal marker) in As_2_O_3_-treated heart tissues. Co-expression of the endothelial marker CD31 with mesenchymal marker α-SMA or FSP1 indicates the intermediate stage of EndMT^41^,^42^. Our observations suggested the participation of EndMT in As_2_O_3_-induced cardiac fibrosis. Meanwhile, we found that As_2_O_3_ induced endothelial cells undergo morphological changes in a concentration-dependent manner, but the changes were mostly visible at a concentration of 8 μmol/l. Moreover, the expression of VE-cadherin and CD31 was decreased and the expression of α-SMA and FSP1 was increased, which indicates the occurrence of EndMT. Nevertheless, the morphological change of the HAECs was not obvious at an As_2_O_3_ concentration of 2 μmol/l. However, when we prolonged the treatment time to 48 h and 72 h, the phenotypic change of the endothelial cells became apparent.

The AKT/ GSK-3β/Snail pathway is an important pathway involved in the process of EMT^30^,^43^. EndMT shares some similarities with EMT including the underlying mechanisms. However, it remains elusive whether this pathway participates in the process of EndMT. As a transcription factor that suppresses cell adhesion, snail has been widely studied in EMT and EndMT. GSK-3β kinase phosphorylation was once thought to be a crucial event during EndMT^44^. Snail can induce EndMT when the activity of GSK-3β is inhibited by PI3K signaling or with LiCl^45^. Our study demonstrated that As_2_O_3_ treatment (8 μmol/l) activated the AKT/GSK-3β/snail pathway in HAECs, and this phenomenon could be prevented by the PI3K inhibitor LY294002. What is more, LY294002 could abolish the process of EndMT induced by As_2_O_3_, which suggests that the AKT/GSK-3β/Snail signaling pathway is also involved in promoting EndMT. Our study did not exclude critical roles of other transcriptional factors and signaling pathways in the process of As_2_O_3_-induced EndMT. And also, it must be noted that the effect of PI3K/AKT inhibitor was detected only in cultured endothelial cells, the results may not be directly applicable to *in vivo* conditions. The *in vivo* role of the AKT/GSK-3β/Snail signaling pathway remains to be explored in the future.

It has been reported that As_2_O_3_ is capable of producing reactive oxide species (ROS)^46^, which might be responsible for the activation of PI3K/AKT. Our results in Supplementary Figure 4 demonstrated this point. But much more detailed studies are needed to further demonstrate the role of ROS in the process of EndMT.

Overall, our present study unraveled that As_2_O_3_ can induce EndMT in HAECs via the AKT/GSK-3β/Snail pathway and that EndMT might be involved in As_2_O_3_-induced cardiac fibrosis. The results of this study suggest that blocking EndMT may provide a viable therapeutic strategy against As_2_O_3_-induced cardiac fibrosis and cardiac dysfunction.

## Methods

### Animal experiments and ethics statements

Healthy male Wistar rats (200–250 g) were obtained from the Experimental Animal Center of the 2^nd^ Affiliated Hospital of Harbin Medical University (Harbin, China). The rats were housed in a standard animal room with constant temperature (23 ± 1 °C) and humidity (55 ± 5%) and subjected to a 12-h light-dark cycle. The rats were randomly divided into four groups: Control, As_2_O_3_ (0.4 mg/kg, 0.8 mg/kg, 1.6 mg/kg). As_2_O_3_ was provided by Harbin YI-DA Pharmaceutical Limited Company and administered intravenously every other day for two weeks. The dosage used in this study was based on previous studies^7^,^47^. The animal experiments were performed under the guidelines from Directive 2010/63/EU of the European Parliament on the protection of animals used for scientific purposes. All the procedures were approved by the Institutional Animal Care and Use Committee of the Harbin Medical University, China [SYXK 2011-033 (2011.12.13 –2016.12.12)].

### Echocardiography

After two weeks of As_2_O_3_ administration, all of the rats were anesthetized with sodium pentobarbital (40 mg/kg, i.p.). The absence of withdrawal reflex to tail pinch was used for monitoring of the adequacy of anaesthesia. After that the rats were prepared for detection of cardiac function with an ultrasound machine (Vivid 7, GE Medical System, USA) as previously described^48^. The following parameters were obtained: interventricular septum (IVS), left ventricular internal dimension (LVID), left ventricular posterior wall (LVPW), ejection fraction (EF), fractional shortening (FS), left ventricular end-diastolic volume (LVEDV), left ventricular mass (LV mass), Left Ventricular end-systolic Volume (LVESV) and the ratio of peak early diastolic ventricular filling velocity to peak atrial filling velocity (E/A).

### Evaluation of collagen deposition

Masson’s trichrome staining and transmission electron microscopy were applied to observe collagen deposition as described previously^49^. Biriefly, following echocardiographic recordings, rats were euthanized by cervical dislocation method for heart tissues, which were cut into different parts. One part was fixed in 4% paraformaldehyde and then embedded in paraffin and sliced cross-sectionally into 5 μm sections for Masson’s trichrome. Masson’s trichrome staining was examined by light microscopy and the images were analyzed with Image Pro Plus software to quantify collagen percentage. For transmission electron microscopy, specimens were prepared and processed by routine methods as previously described^7^.

### Cell culture

Primary HAECs were purchased from Sciencell (No. 6100) and maintained in collagen-coated flasks with endothelial basal medium (ECM, Sciencell, No. 1001). The cells were cultured at 37 °C with 5% CO_2_, and the medium was changed every 48 hours. When the culture reached 70% confluence, the cells were treated with various concentrations of As_2_O_3_ (2, 4 and 8 μM) for 24 h. The PI3K inhibitor LY294002 was obtained from Cayman chemical company (Ann Arbor, Michigan, USA), which was used to inhibit the phosphorylation of AKT in HAECs at a concentration of 20 μM.

### RNA extraction and real-time quantitative RT-PCR (qRT-PCR)

Total RNA was extracted from cardiac tissues and HAECs using TRIZOL reagent (Invitrogen, USA) as previously described^50^,^51^, and then it was reverse transcribed into cDNA with a High-Capacity cDNA Reverse Transcription Kit (Applied Biosystems, Foster City, CA, USA) according to the manufacturer’s instructions. Real-time PCR was performed to determine mRNA expression levels with an ABI 7500 fast Real Time PCR system (Applied Biosystems, USA). GAPDH was used as an internal control. The sequences of the primers are presented in Supplementary Table 1.

### Protein extraction and western blot analysis

Protein samples were extracted from tissues and cells with the same procedures described previously^52^,^53^. Briefly, heart tissues and HAECs were lysed in RIPA buffer and then centrifuged at 4 °C 13500 rpm for 15 minutes. The supernatant was collected, and protein concentrations were determined by bicinchoninic acid (BCA) protein assay (Beyotime, Shanghai, China). Protein extracts were separated by SDS–PAGE and transferred onto a nitrocellulose membrane. The membranes were blocked and incubated with the following primary antibodies: anti-VE-cadherin antibody, anti-CD31 antibody, anti-α-SMA antibody, anti-FSP1 antibody, anti-Snail antibody (Abcam, Cambridge, MA, USA), anti-p-AKT antibody, anti-AKT antibody, anti-p-GSK-3β antibody, anti-GSK-3β antibody (Wanleibio. Shenyang, China), and anti-GAPDH antibody (Kangcheng, Shanghai, China); GAPDH was used as a loading control. After incubation at 4 °C overnight, the membrane was washed three times with PBST, incubated with fluorescence-conjugated goat anti-rabbit IgG and goat anti-mouse IgG for one hour at room temperature (1:10000, Invitrogen) and scanned by Odyssey Imaging System (LI-COR, Inc., Lincoln, NE, USA).

### Immunofluorescence

Immunofluorescence staining was performed to detect the expression of CD31 and α-SMA in HAECs. Briefly, the cells were cultured on coverslips and received the desired treatment. At the end of the treatment, the cells were washed with PBS and processed in the same way as previously described^38^. For *in vivo* study, frozen heart tissues were cut into 5 μm thick sections to detect the phenomenon of EndMT. The secondary antibodies conjugated with Alexa Fluor 594 and Alexa Fluor-488 (Invitrogen, Carlsbad, CA, USA) were used in this experiment. Double positive labeling of CD31 and α-SMA was regarded as cell transformation. The immunofluorescence-labeled cells and tissues were examined and analyzed by laser scanning confocal microscopy (FV300, Olympus, Japan).

### Statistical analysis

Results are expressed as mean ± SEM and were analyzed by one-way analysis of variance (ANOVA) with GraphPad Prism 5.0 software. P < 0.05 was considered statistically significant.

## Additional Information

**How to cite this article**: Zhang, Y. *et al*. Endothelial to mesenchymal transition contributes to arsenic-trioxide-induced cardiac fibrosis. *Sci. Rep*. **6**, 33787; doi: 10.1038/srep33787 (2016).

## Supplementary Material

Supplementary Information
