# Supplementary material for: Endothelial to mesenchymal transition contributes to arsenic-trioxide-induced cardiac fibrosis
Source: Sci Rep. 2016 Sep 27;6:33787. doi: 10.1038/srep33787 (PMC5037371; doi:10.1038/srep33787)

Supplementary materials

Endothelial to mesenchymal transition contributes to arsenic-trioxide-induced cardiac fibrosis

Yong Zhang[1,2*#], Xianxian Wu[1*], Yang Li[3*], Haiying Zhang[1], Zhange Li[1], Ying Zhang[1], Longyin Zhang[1], Jiaming Ju[1], Xin Liu[1], Xiaohui Chen[1], Peter V. Glybochko[4], Vladimir Nikolenko[4], Philipp Kopylov[5], Chaoqian Xu[1], Baofeng Yang[1,6#]

[1]Department of Pharmacology (the State-Province Key Laboratories of Biomedicine-Pharmaceutics of China, Key Laboratory of Cardiovascular Research, Ministry of Education), College of Pharmacy, Harbin Medical University, Harbin, 150081, China

[2]Institute of Metabolic Disease, Heilongjiang Academy of Medical Science, Harbin, 150086, China

[3]Center for Endemic Disease Control, Chinese Center for Disease Control and Prevention, Key Lab of Etiology and Epidemiology, Education Bureau of Heilongjiang Province & Ministry of Health (23618504), Harbin Medical University, Harbin, 150081,China

[4]The Research Center, Sechenov First Moscow State Medical University, Moscow, 119991, Russia

[5]Department of preventive and emergency cardiology, Sechenov First Moscow State Medical University, Moscow, 119991, Russia

[6]Department of Pharmacology and Therapeutics, Melbourne School of Biomedical Sciences, Faculty of Medicine, Dentistry and Health Sciences, The University of Melbourne, Melbourne, 3010, Australia

*Yong Zhang, Xianxian Wu and Yang Li contributed equally to this study.

[#]Corresponding author at: Department of Pharmacology, College of Pharmacy, Harbin Medical University, Harbin, 150081, China. E-mail: hmuzhangyong@hotmail.com; yangbf@ems.hrbmu.edu.cn

Supplementary Tables

Supplementary Table 1. Primers used for real-time qPCR (h and r indicate human and rat species, respectively).

Gene	Primer Sequences (forward, reverse)
VE-cadherin (h)	F: 5’- CAGCCCAAAGTGTGTGAGAA-3’<br>R: 5’-TGTGATGTTGGCCGTGTTAT-3’
VE-cadherin (r)	F: 5’-ACCAGTGACAGAGGCCAATACT-3’<br>R: 5’- GGCCTCCACAGTCAGGTTATAC-3’
CD31(h)	F: 5’- GAGTCCAGCCGCATATCC-3’<br>R: 5’-TGACACAATCGTATCTTCCTTC-3’
CD31(r)	F: 5’-CTCCTAAGAGCAAAGAGCAACTTC-3’<br>R: 5’-TACACTGGTATTCCATGTCTCTGG-3’
α-SMA(h)	F: 5’-TGACAATGGCTCTGGGCTCTGTAA-3’<br>R: 5’-TTCGTCACCCACGTAGCTGTCTTT-3’
α-SMA(r)	F: 5'-CTGTGCTATGTCGCTCTGGA-3’<br>R: 5'-CTGTGCTATGTCGCTCTGGA-3’
FSP1(h)	F: 5’-GTCCACCTTCCACAAGTAC-3’<br>R: 5’-TGTCCAAGTTGCTCATCAG-3’
FSP1(r)	F: 5’- AGGACAGACGAAGCTGCATT-3’<br>R: 5’- CTCACAGCCAACATGGAAGA-3’
Snail(h)	F: 5’-GCCTTCAACTGCAAATACTGC-3’<br>R: 5’- CTTCTTGACATCTGAGTGGGTC-3’
Col1a(h)	F: 5’- CTTGCTTGAAGACCCATGCG-3’<br>R: 5’- TTGGCAGTCTGAGAACCCCA-3’
Col1a(r)	F: 5’-GGTCAGACCTGTGTGTTCCC-3’<br>R: 5’-GGTCCATGTAGGCTACGCTG-3’
Col3a(h)	F: 5’- AACACGCAAGGCTGTGAGACT-3’<br>R: 5’-TTTTGTCGGTCACTTGCACTG-3’
Col3a(r)	F: 5’-CAATGTAAAGAAGTCTCTGAAG-3’<br>R: 5’-CAAACAGGGCCAATGTCCAC-3’
Mmp2(h)	F: 5’- CCGTCGCCCATCATCAA-3’<br>R: 5’-AGATATTGCACTGCCAACTCT-3’
Mmp2(r)	F: 5’-TCCCGAGATCTGCAAGCAAG-3’<br>R: 5’-AGAATGTGGCCACCAGCAAG-3’
Mmp9(h)	F: 5’-GTGCTGGGCTGCTGCTTTGCTG-3’<br>R: 5’-GTCGCCCTCAAAGGTTTGGAAT-3’
Mmp9(r)	F: 5’-TGGAACTCACACAACGTCTTTCA-3’<br>R: 5’-TCACCCGGTTGTGGAAACTC-3’
Fibronectin(h)	F: 5’- GATAAATCAACAGTGGGAGC-3’<br>R: 5’-CCCAGATCATGGAGTCTTTA-3’
Fibronectin(r)	F: 5’-TCAGCTGTACCATTGCAAATC-3’<br>R: 5’-TGGTGTCCTGATCATTGCAT-3’
Vimentin(h)	F: 5’-GAGAACTTTGCCGTTGAAGC-3’

	R: 5'-TCCAGCAGCTTCCTGTAGGT-3'
Vimentin(r)	F: 5'-TCCCTGAACCTGAGAGAAAC-3'<br>R: 5'-ATCGTGGTGCTGAGAAGTC-3'
GAPDH(h, r)	F: 5'-AAGGTCGGAGTCAACGGATTT-3'<br>R: 5'- AGATGATGACCCTTTTGGCTC-3'

Supplementary Table 2. The parameters of echocardiography of rats after exposure to As_2O_3.

	Control	As_2O_3 (0.4 mg/kg)	As_2O_3 (0.8 mg/kg)	As_2O_3 (1.6 mg/kg)
IVS;d(mm)	1.84 ±0.10	1.89 ±0.05	1.99 ±0.04	2.23 ±0.14*
IVS;s(mm)	2.72 ±0.09	2.90 ±0.09	3.16 ±0.10	3.21 ±0.28
LVID;d(mm)	5.07 ±0.09	6.72 ±0.18**	6.74 ±0.38**	6.88 ±0.31**
LVID;s(mm)	2.69 ±0.09	3.84 ±0.14*	3.90 ±0.44*	4.46 ±0.25**
LVPW;d(mm)	1.79 ±0.06	2.03 ±0.06	2.17 ±0.10*	2.39 ±0.11**
LVPW;s(mm)	2.80 ±0.03	2.93 ±0.08	3.00 ±0.09	3.18 ±0.07**
LV mass(mg)	666.5 ±43.40	932.6±31.77	975.1 ±60.64*	1039.0 ±120.70*
LVEDV(μL)	145.5 ±8.53	238.1 ±10.26	238.2±32.13*	244.0 ±27.15*
LVESV(μL)	35.17 ±11.66	77.20 ±4.05	83.43 ±14.34*	100.50 ±13.21**

Results represent mean ± S.E.M. n = 4–5 rats in each group. IVS, interventricular septum; LVID, left ventricular internal dimension; LVPW, left ventricular posterior wall; LV mass, left ventricular mass; d, diastole; s, systole; LVEDV, left ventricular end-diastolic volume; LVESV, Left ventricular end-systolic volume.

Supplementary Figures

Supplementary Figure 1. Collagen production in the control group and As_2O_3-treated groups by transmission electron microscopy. Collagens are indicated by yellow stars. Scale bar=2 μm. *p < 0.05 vs Control. n=3.

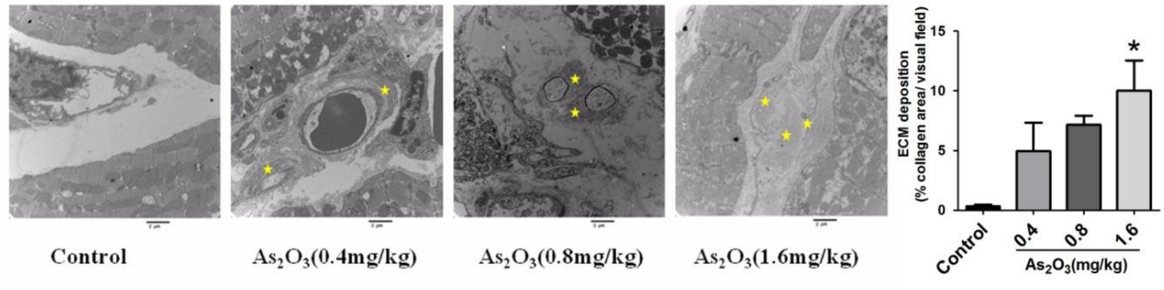

Supplementary Figure 2. Relative mRNA expression of EndMT marker genes SNAIL, TWIST and SLUG in heart tissue. *p<0.05, **p<0.01, ***p<0.001 vs Control. n=3.

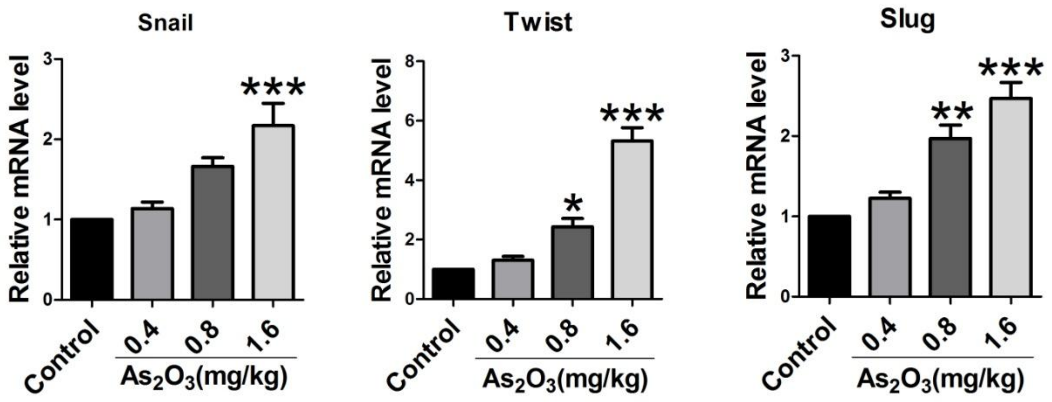

Supplementary Figure 3. Effects of As_2O_3 on the AKT/GSK-3β/Snail signaling pathway in vivo. (a)The protein levels of p-AKT, AKT, p-GSK-3β, GSK-3β and Snail were detected by western blotting in heart tissues after exposure to As_2O_3. n=3. (b) Co-immunostaining of CD31 and p-AKT in cardiac sections of rats treated with or without As_2O_3, nuclei are stained with DAPI (blue). Scale bar=30 μm. (c) Co-immunostaining of CD31 and p-GSK-3β in cardiac sections of rats treated with or without As_2O_3, nuclei are stained with DAPI (blue). Scale bar=30 μm. (d) Co-immunostaining of CD31 and snail in cardiac sections of rats treated with or without As_2O_3, nuclei are stained with DAPI (blue). Scale bar=30 μm.

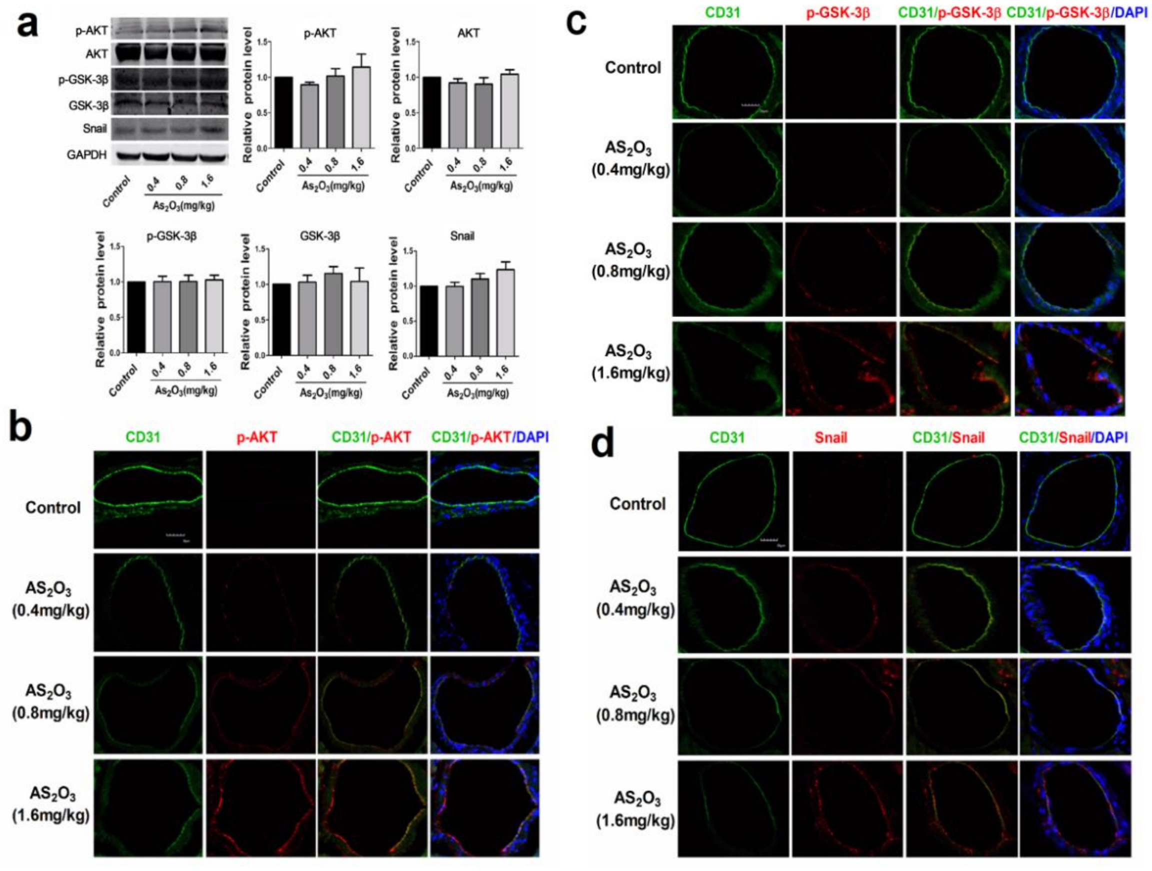

Supplementary Figure 4. Inhibitory effect of NAC on intracellular ROS accumulation and on the activation of AKT/GSK-3β/Snail pathway when exposed to As_2O_3. (a) Confocal images of 2',7'-dichlorofluorescein (DCF) staining showed ROS level in control endothelial cells, cells incubated with As_2O_3 and cells in the presence of As_2O_3(8 μM) and NAC (5 mM). (b) Representative western blotting bands and analyzed data for the expression of p-AKT, AKT, p-GSK-3β, GSK-3β and snail in endothelial cells in different groups. *$p<0.05$, **$p<0.01$, ***$p<0.001$ vs Control, $^{\#}p<0.05$, $^{\#\#}p<0.01$, $^{\#\#\#}p<0.001$ vs As_2O_3. n=3.

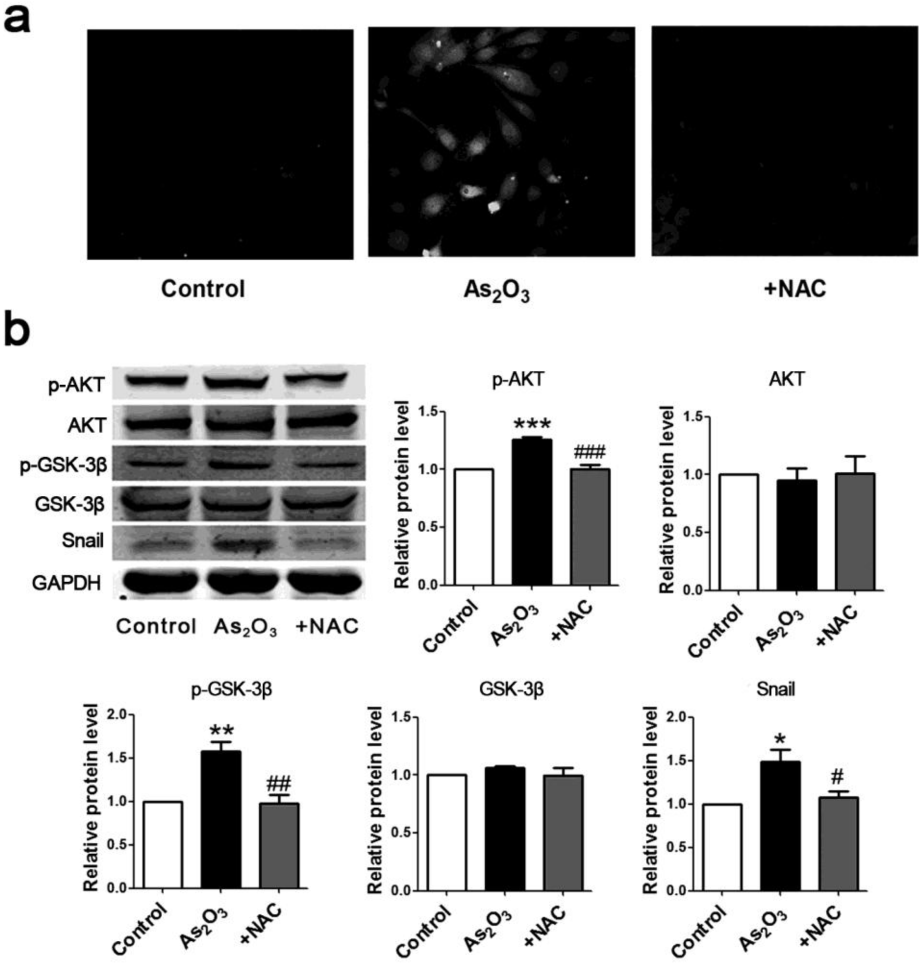

Supplement: Supplementary Information [file srep33787-s1.pdf]
